# Striking back against racist violence in the East End of London, 1968–1970

**DOI:** 10.1177/0306396816642997

**Published:** 2016-06-24

**Authors:** Stephen Ashe, Satnam Virdee, Laurence Brown

**Keywords:** autonomous self-organisation, Enoch Powell 1968, integration, Paki-bashing, Pakistani organisations, Peter Shore MP, policing, racist violence and harassment, self-defence, Tosir Ali, Tower Hamlets

## Abstract

This article tells the hitherto untold story of how different Pakistani organisations mobilised in response to racist violence and harassment in the east London Borough of Tower Hamlets (1968–1970). In telling this story, the authors analyse the problematic nature of official and public understandings of, and responses to, racist violence, and how it distorted the lives of racialised minorities. Drawing on original archival research carried out in 2014, this piece identifies the emergence of two distinct political repertoires from within the Pakistani community: the integrationist approach and the autonomous approach. The integrationist approach involving the Pakistani Welfare Association (PWA) and the National Federation of Pakistani Associations (NFPA) tried to address the problem through existing local state ‘race relations’ apparatuses and mainstream political channels, while at the same time re-establishing consent for the police as the agents of law and order. In contrast, a network of Black Power groups, anti-imperialists and socialists led by the Pakistani Progressive Party (PPP) and the Pakistani Workers’ Union (PWU) challenged both the local political leadership and the authority of the police in Tower Hamlets, while also undermining the stereotype of Asian people as ‘weak’ and ‘passive’. In recovering this lost episode of resistance to ‘Paki-bashing’, unleashed in the aftermath of Enoch Powell’s inflammatory speeches, this essay makes a contribution to the history of autonomous anti-racist collective action undertaken by racialised minorities in Britain.

**Figure 1.**
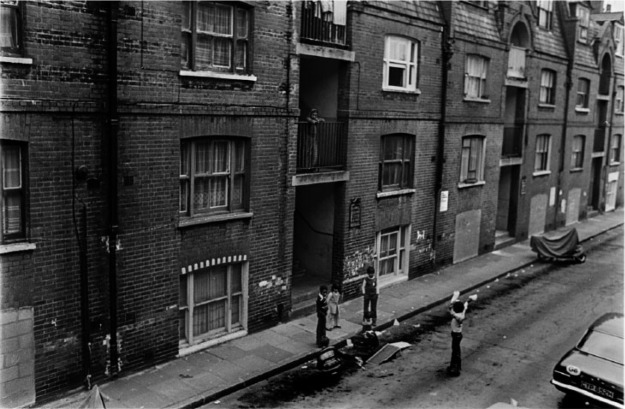
Fieldgate Mansions, Spitalfields , Tower Hamlets (image courtesy of David Hoffman | www.hoffmanphotos.com/)

From the violent anti-black riots in Nottingham and Notting Hill in the 1950s to the continuing racist attacks throughout British towns and cities today, racist violence has been a constant feature of Britain’s post-war social and political landscape. At the same time, racialised minorities have never been merely the ‘passive objects’ of such racism.^1^ There is a long history of racialised minorities collectively fighting back against violence and harassment through physical resistance, political mobilisation and cultural action.^2^ Recent research has focused on the distinct social movements, based in second-generation youth, against constant racial harassment (including by the police) at a time when the National Front was gaining support during the mid-1970s.^3^ This archival research explores the antecedents of those campaigns through analysing the mobilisation by Pakistani organisations and their allies against a rising tide of racist violence and harassment in the East End of London between 1968 and 1970. This story of resistance, which hitherto has largely been marginalised in the existing literature,^4^ provides important insights into solidarity networks at the time and the two distinct political repertoires that emerged in Tower Hamlets in response to the issue.

Experiences of, and responses to, racist violence and harassment were fiercely contested in the 1970s, as racialised minority groups struggled to get the criminal justice system and other state agencies to accept the severity of the problem.^5^ It was only in the 1980s and 1990s – in part due to the entry of Black and Asian people into academic life – that academics finally began to focus their attention on this particular form of racism. Subsequent studies have drawn attention to the scale of the problem and how police statistics and state-sponsored surveys have underestimated its prevalence.^6^ These studies have also highlighted the ways in which the victims of attacks have been accused of fabricating and exaggerating the extent of racist violence as well as the systemic failures of, and harassment by, state authorities. At the same time, the police have tended to reduce the status of racist attacks to that of ordinary criminal assaults, thus rendering racism insignificant.^7^ Academic research has also drawn attention to the ways in which such violence distorts the everyday lives of racialised minorities, including forcing them to make adjustments in their daily routines just so they can minimise the risk of being attacked.^8^ And, relatedly, recent research has demonstrated the detrimental consequences of such racism on peoples’ long-term physical and mental health.^9^ In contrast to this body of research, official and public understandings of racist violence and harassment have tended to eschew any proper discussion of wider ‘societal racism’.^10^ As a result, such violence has been commonly misunderstood as occurring in a vacuum, detached from structural and institutional racism. The effect of this separation from a wider context is that racist violence and harassment is typically portrayed as being the sport of choice for the extreme Right or an anomic and lunatic minority, while at the same time being framed as something that is random, sporadic, unpredictable and opportunistic. We, on the other hand, contend that racist violence is underpinned by a ‘territorial logic’ that seeks to ‘expunge those “others” from the “white terrain”’.^11^ It is our view that recurring verbal abuse, threatening and intimidating behaviour, malicious complaints, violence, property damage and even murders are not at all random: the victims of racist violence and harassment are attacked specifically because they are considered to be a threat to an imagined white British society.^12^ Such imagining is of course enhanced each time immigration is discussed in hostile terms, as happened for example in 1968 during what became known as the ‘Kenyan Asian Affair’ and the climate provoked by Enoch Powell’s ominous speech about ‘Rivers of Blood’. At the same time, extensive legal and policy frameworks help to sustain the notion of a ‘tolerant British culture’.^13^ This is a perception partly built upon the occlusion of the cumulative patterns of racist violence and harassment, and any proper discussion of the political contexts within which these patterns emerge.

Whereas existing scholarship has mostly focused on racialised minorities as victims or on the extreme Right as agents of violence, much less attention has been given to how racist violence often provides the impetus for political action, including different forms of mobilisation and coalition-building. The ways in which different groups recorded their own data on racist attacks play an important role in challenging official and public understandings by shifting the point of discussion away from racist violence as an exceptional event to demonstrating that it is part of a more pervasive experience of racism. This form of resistance is all the more important in light of the fact that most attacks are not reported to the police for fear of recrimination or because the police themselves have been the agents of such violence and harassment.^14^ And it is against this backdrop that we draw attention to the different networks, strategies and ideological standpoints employed by Pakistani organisations in response to the rising level of racist violence in the East End of London between 1968 and 1970.

This article offers a detailed analysis of two distinct responses to violence and harassment: the integrationist approach and the autonomous approach. Distinct ideological differences aside, what distinguishes them is the way in which they interacted with and related to the state and their respective positions on the question of self-defence. On the one hand, the integrationist approach sought to forge a collaborative relationship between the political establishment, existing state structures and sections of civil society, and thereby assimilate the recently settled Pakistani migrant community into the so-called ‘British values’ of law and order and the authority of the police. On the other hand, the autonomous approach was part of a wider network of collective anti-racist action that was increasingly bypassing the state, mainstream party politics and the apparatuses of the race relations industry. These two counterposed positions, described in detail below, were not only to surface again when taking on the threat of the extreme Right, for example, in Lewisham in 1977 and Southall in 1979, but were to underlie many of the deep contradictions which characterised community politics across Britain during the 1980s.

The 1968 Race Relations Act had transformed the recently created National Committee for Commonwealth Immigrants into a national Community Relations Commission (CRC) ‘to promote harmonious community relations’. As well as creating new local committees, the CRC sought to coordinate and ‘manage’ local groups already in existence such as the Citizens Committee of Tower Hamlets (CCoTH) based at Toynbee Hall. The integrationist approach was backed by the local Member of Parliament, local state apparatuses such as the CCoTH and civil society organisations including the Pakistani Welfare Association (PWA) and the National Federation of Pakistani Associations (NFPA), which used conventional campaign methods in their attempt to move the political establishment and other sections of local civil society towards a better understanding of just how precarious everyday life had become for the local Pakistani population. Our research shows how this approach reinforced many of the presuppositions underpinning official and public conceptions of racist violence, and implicitly helped to re-secure consent for the authority of the police and the traditional values of ‘law and order’. In contrast, the Pakistani Progressive Party (PPP) and the Pakistani Workers’ Union (PWU) spearheaded a multiethnic coalition of Black Power groups, anti-imperialists and socialists. While challenging the authority of the police, the PPP, PWU and their allies also challenged the authority of the established political leadership in Tower Hamlets at both local state and civil society levels. They did so by offering an altogether different interpretation of the nature and scale of racist violence and harassment and by calling for self-defence measures. In making this call, this coalition emphatically countered the racist belief that Pakistani people were ‘passive objects’^15^ and ‘unwilling to fight back’.^16^

## Background to the study: why Tower Hamlets?

We have chosen to focus on events in Tower Hamlets for several reasons. First and foremost, events in Tower Hamlets between 1968 and 1970 captured the attention of the national media. Further, focusing on this moment where developments in the local area became a national event enables us to tell the relatively untold story of Pakistani responses to racist violence. And telling this story makes an important contribution to the way in which the politics of migration, racism, fascist political agitation, anti-racism and anti-fascism in the East End of London have long been a key focus of study in Britain.

The London Borough of Tower Hamlets forms the core of the traditional East End including areas such as Whitechapel, Stepney, Bethnal Green, Poplar and Shoreditch. Ever since the formation of the East India Company in the sixteenth century, Tower Hamlets has been the gateway through which people, as well as goods, from around the world came to make their first acquaintance with England, and then Britain.^17^ Irish Catholics began settling in parts of the East End as early as the eighteenth century while Jewish migrants escaping the racist pogroms in the Tsarist Empire made their home there from the late nineteenth century. Irregular work, subsistence-like living conditions, combined with their location – barely a mile away from some of London’s wealthiest districts – helped to produce a multiethnic working class that could quickly turn towards, as well as lead, transformative working-class struggles. But there was also an underside of racism and anti-Semitism that was ever-present in the East End, and often led to outbreaks of co-ordinated racist violence directed against those workers who could not be imagined as British. From the Gordon Riots of 1780 where the British ‘Church and King mob’ rampaged through Spitalfields burning the homes of Catholics to the attempts in the 1930s by Oswald Mosley and the British Union of Fascists to physically quash what they termed to be the ‘Jewish menace’, racism has been an integral and powerful structuring force in the lives of ordinary ‘Eastenders’.^18^ Indeed, migration and racist violence continued to be a feature of the East End’s post-war landscape.^19^

By the 1960s, migration from Britain’s colonial territories meant that the local population of Tower Hamlets included around 3,000 people from the Caribbean and 8,000 ‘Asian’ people (of whom 4,300 were from East Pakistan – now Bangladesh).^20^ In fact, 95 per cent of Sylheti Pakistanis were men, who had come in advance of their families. Finding work in the rag-trade and later catering, many of the new migrants who settled around the Brick Lane area were forced to reside in small tenement blocks in the poorest areas in the east of Tower Hamlets. Their social and economic precarity was worsened by intensifying racist violence during the late 1960s. A survey carried out by the Pakistani Student Federation in April 1970 revealed that more than one-quarter of their members had been physically assaulted in the last year.^21^ A CRC report also noted that there had been at least forty-three separate incidents including property damage, robbery and molestation during the first three months of 1970.^22^ In the same month, a local youth worker reported that ‘possibly the worst racial problem’ in Tower Hamlets ‘is considerable “Pakibaiting” and “rolling” (robbing with violence) by some of the local young people. The situation is becoming both violent and unhealthy and is evident in schools as well as the streets.’^23^

This escalation in racist violence culminated in Tosir Ali, a 50-year-old kitchen porter and father of four, being brutally stabbed in the St Leonard Street area of Tower Hamlets on 6 April 1970.^24^ After being attacked, Mr Ali managed to make his way home, but later died in hospital from his injuries.^25^

## From the ‘politics of convention’ to the ‘politics of the street’

No doubt much of the violence being meted out by white gangs on a regular basis was freelance and opportunist but the anti-immigrant atmosphere was constantly being reinforced by extremist groups in the area. During the mid-1960s, a number of fascist, racist nationalist and national socialists started working together, culminating in the formation of the National Front (NF) on 7 February 1967. Its mission was to oppose immigration, multicultural policies and to maintain white privilege in all spheres of economic, political and cultural life. On 20 April 1968, Enoch Powell delivered his infamous ‘Rivers of Blood’ speech to an audience of Conservatives in Birmingham. Employing emotive anecdotes, Powell provocatively warned of the alleged dangers that ‘black migration’ posed to ‘white Britain’. In fact, Powell prophesied that ‘black immigration’ represented impending lawlessness and anomie.^26^ While Powell was condemned by most of the political class, including much of his own party leadership, consecutive Labour and Conservative governments went on to introduce the 1968 and 1971 Immigration Acts to control ‘black immigration’. At the same time, Powell’s speech received considerable working-class support, particularly in the East End of London. On 23 April 1968, just three days after the speech, 1,000 dockers from the West India Dock in Poplar struck in protest against Powell’s sacking from his position as shadow defence minister. Some of them marched from the East End to Westminster carrying placards saying ‘Back Britain, not Black Britain’. Three days later, some 4,400 men refused to go into work at the Royal Group of Docks in Newham. Harry Pennington, leader of the strike, demanded a total ban on immigration, claiming that the ‘full weight of any influx of coloured people’ was felt most strongly in the Docklands area.^27^ Overall, about a third of the registered labour force of dockworkers – between 6,000 and 7,000 men – was involved in strike action in the week and, to add fuel to the fire, 600 meat porters from Smithfield market in the East End struck and marched to Westminster handing Powell a ninety-two page petition supporting his stance.^28^

This shift towards the ‘politics of the street’ was not confined to working-class support for Powell. Between 1965 and 1970, various Asian and Black political organisations were moving away from the dominant liberal, state integrationist model with its belief that, given compromise on both sides, Britain’s institutions and structures could be reformed. Inspired by Black Power, anti-colonial struggles and radical left-wing politics, many organisations were now bypassing the state, mainstream party politics and the organs of the ‘race relations industry’ such as the National Committee for Commonwealth Immigrants and the Race Relations Board, which, as Sivanandan has pointed out, were regarded as a means of socially controlling black resistance.^29^ West Indian and Asian activists were now appropriating the ascribed identity of black and infusing it with a new ideological meaning in a bid to fashion powerful ‘communities of resistance’.^30^

One of the casualties of the new-found militancy was the organisation CARD (Campaign Against Racial Discrimination). Formed at the instigation of Martin Luther King who had visited London in December 1964, CARD was a federation of various Asian and West Indian groupings alongside Labour Party campaigners lobbying for anti-discrimination laws. On the one hand, the state race-relations bodies were luring away its leading lights; on the other hand, militant black activists wanted little to do with its liberal programme. This was reflected in the formation of the Radical Action Adjustment Society, after Malcolm X’s visit to England in 1965, which declared: ‘Black men, unite … we have nothing to lose but our fears.’^31^ Thus by 1967, the West Indian Standing Conference, National Federation of Pakistani Associations and ‘more militant’ black activists had left CARD and it was wound up soon after.^32^ It is important to note that the kind of tension within CARD, between those who wanted to work within ‘the system’ and those who wanted a riposte to racism that was independent of the state,^33^ was very similar to that between the integrationist and the autonomous approaches to combating racial violence in Tower Hamlets in 1968–1970.

The late 1960s was to witness a whole range of new black organisations. In recognition of ‘the need to fight both imperialism and racism’, the Universal Coloured People’s Association (UCPA) was established in 1967.^34^ As well as organising a Black Power rally in Brixton, the UCPA and the Caribbean Workers’ Movement began collecting data and fighting cases of police brutality in London and Manchester.^35^ In April 1968, on the very same day that the dockers and porters marched in support of Powell, representatives from more than fifty organisations came together to form the Black People’s Alliance (BPA), a ‘militant front for black consciousness and against racialism’. It refused membership to any organisation that had ‘compromised with government policy … fallen prey to government hand-outs … or looked to the Labour Party for redress’.^36^ Moreover, concerned with the rising levels of racist violence and harassment, a lack of police protection and police intimidation, those present at the formation of the BPA also pledged to make the necessary ‘preparations for protection and security’ and the need to form ‘vigilante patrols’.^37^

South Asian political formations were also being shaped by Black Power, anti-colonial struggles and left-wing political traditions. Hence, the politics of organisations such as the PPP and the PWU were in part informed by political developments in Naxalbari in West Bengal and events leading up to the Bangladesh War of Independence in 1971.^38^ However, shared experiences of racism in Britain and imperialism also led to the PPP and the PWU working side-by-side with other anti-imperialists who were arguing that any response to racism could not be divorced from an understanding of Britain’s imperial project.^39^ The PPP and the PWU were also part of a broader network of resistance. Almost immediately after Powell’s speech, the PWU, CARD and the Working People’s Party of England (WPPE) began collating data on racist attacks in London.^40^ Less than a month after the formation of the BPA, at a meeting held in Camden in May 1968, some of the ‘Pakistani, West Indian and English people’ in attendance decided that the situation had become so serious that they moved to set up self-defence patrols.^41^ At the same time, the PPP and the PWU began working with the Irish National Liberation Solidarity Front (INLSF). In fact, the WPPE provided office services to several black and liberation movement organisations such as the UCPA, the Black Panthers and the INLSF^42^ – many of which would play an important role in the struggle against racist violence in Tower Hamlets.

## Responses to racist violence in Tower Hamlets, East London

The following section offers a ‘thick description’ of the integrationist and autonomous anti-racist responses to racist violence in Tower Hamlets between 1968 and 1970. The integrationist approach, of which the PWA and the NFPA were central proponents, attempted to make the political establishment more aware of the precarious nature of everyday life for Pakistani people through conventional political methods. A key presupposition was that there were no inherent structural obstacles to both the local and the national state recognising the severity of racist violence – all that was required was that staff become more sensitive and alive to events on the ground. What is more, the integrationist response also echoed many of the official and public conceptions of racist violence discussed earlier. As well as downplaying the fact that racist violence had become part of the everyday lived reality for Pakistani women, men and children, this approach contributed to the reproduction of the racist stereotype of Asian people more generally as being ‘meek’ and ‘submissive’. The discussion below also draws attention to the way in which the integrationist approach conceptualised racist violence in class terms. Specifically this involved attempts to equate racism with rowdy, troublesome and socially deprived youths, ‘skinheads’ and ‘hooligans’, while at the same time separating racist violence and harassment from both institutional racism and the more ‘polite’, ‘genteel’ racism of the middle and political classes. In this integrationist response there was, as Paul Gilroy wrote in another context, ‘no moral panic about racist violence’.^43^

By contrast, African, Asian, Caribbean and Irish political activists and a small number of white English socialists saw the need for immediate and direct action, resulting in the formation of a multiethnic anti-racist network. Drawing on their existing links, the PPP- and PWU-led approach revealed a more nuanced understanding of the nature and scale of racist violence and harassment – one which was grounded in a political understanding of Britain’s role as an imperialist power. What is more, these groups gradually bypassed ‘the politics of convention’ in favour of the ‘the politics of the street’.^44^ By emphasising the need for self-defence tactics, the PPP, PWU and their allies challenged prevailing views of law and order, the authority of the police, the leadership of the political establishment and the racist suggestion that Pakistanis were ‘meek’ and ‘submissive’. The move towards self-defence tactics also contested the state’s responsibility to protect ‘the public’ from racist violence and harassment. Drawing attention to the severity of racism and the failures of the state, the autonomous approach played an important precursory role in what later became concerted calls to recognise the collective right to self-defence.^45^

### The integrationist response to racist violence

Soon after Powell’s ‘Rivers of Blood’ speech, Joseph Hunte,^46^ Chairman of the CCoTH, informed the police that the situation regarding racist violence in Tower Hamlets ‘was becoming serious’.^47^ In response to the escalation in such violence, the CCoTH established an ‘investigation clinic’ to explore its ‘root cause’ and ‘to give the Pakistani community assurance[s] that efforts are being made to prevent further incidents’.^48^ Working alongside local churches, synagogues, mosques, youth and community workers, teachers and personnel firms, the CCoTH set out to ‘work with the police in any matter relating to community problems’ and to be ‘a panel through which guidance could be given to any member of the community to assist them in getting justice for any act of violence against them’.^49^ The ‘investigation clinic’ also had a clear educative function, namely to help ‘the Asian community … understand the role of the police by carefully examining incidents they have reported to the police and seeing that such reports are properly dealt with as far as the powers of the police allow’.^50^

In other words, the ‘investigation clinic’ was responsible for implementing an assimilatory agenda and securing consent for the police’s ‘way of doing things’. However, the idea that the local ‘Asian community’ had to be educated with regard to the role of the police was a message that was repeatedly reinforced in highly racialising terms. For example, Chief Inspector Ernest Roberts, ‘the police liaison officer for community relations’ in Tower Hamlets, made the following claims:

They still have the village headman idea, and think we are discriminating if we do not immediately find and punish someone after an incident.^51^

And:

These people know the police in India or Pakistan, where they are tougher than we are. So if we don’t act in this tough way, they believe it’s some kind of opting out.^52^

This discourse was also reinforced by Hunte, who seemed to suggest that there was something in the nature of the Pakistani residents that made them more susceptible to racist attacks:

I get the impression that the West Indians are quite liked. Their language and behaviour patterns are the same. But the Paks [sic] are introspective and more remote. And they are never willing to resist. If the skinheads tried it on West Indians they would give them a rough old tumble.^53^

Overall, the integrationist approach to tackling racist violence was underpinned by prejudices that were themselves contributing to the further racialisation of local residents of Pakistani descent. As a result, the integrationists helped to deflect attention away from white racism and towards the need to familiarise racialised minorities about the due process of the law, and the need to ‘fit in’ like other minority groups. This approach was infused with assimilatory, if not colonialist, ‘civilising’ undertones, evidenced in calls for the local Pakistani population to be educated as to the role of the police in the ‘Mother Country’.

The local Labour MP for Stepney, Peter Shore, was responsible in part for the tenor of the integrationist response. The scale of the problem of racist violence was such that Salman Ali – Pakistan’s High Commissioner – was forced to intervene. Immediately after the murder of Tosir Ali on 6 April 1970, Salman Ali visited the East End of London ‘to let the Pakistani community there know that he was very concerned about the attacks on them’.^54^ He also informed journalists that discussions had been held with the CRC and that the matter ‘would be put before the Home Office and the authorities concerned’. The High Commissioner also spoke at a local meeting attended by over 100 people, including Shore, the police and Joseph Hunte. Shortly after Salman Ali’s visit, Shore wrote to the Home Secretary, James Callaghan, on 14 April to inform him that between February and April 1970, there had been at least twenty racist attacks in Tower Hamlets.^55^ In addition, Salman Ali met with the Home Secretary who attempted to reassure the High Commissioner that ‘special efforts’ were being made to ‘seek out and arrest’ the perpetrators of racist violence.^56^ During the same meeting, Callaghan also insisted that ‘the effects of single incidents should not be exaggerated’.

Following a progress report and meetings with representatives of the local Pakistani community, Shore released a press statement claiming that he ‘was confident that the police can handle this problem’ and that he had ‘no doubt of their anxieties and determination to handle it effectively’.^57^ Three days after Shore’s press release, the NFPA called a meeting at the Grand Palace Hall. Attended by Shore, the police and representatives from the PWA, the meeting drew up a ‘five-point programme condemning violence and appealing to the police and local authorities to take action’.^58^ On the 20 April, the national CRC, Shore and Ian Mikardo (MP for the neighbouring constituency of Poplar) held an ‘exploratory meeting’ at the House of Commons, attended by representatives of the PPP and the UCPA.^59^ Despite Shore’s previous public statement of faith in the ability of the police to ‘handle this problem’, the minutes from the meeting noted that ‘There was a feeling that the police have not been fully cooperative in answering calls for help.’^60^

Throughout April 1970, regular local meetings were arranged to create a dialogue between the local MPs, the police, the CCoTH, the Inner London Educational Authority, local churches and representatives from the NFPA and the PWA.^61^ The first meeting was held on Saturday 18 April, shortly after a series of ‘skirmishes’ between young Pakistani men and ‘skinheads’ in Brick Lane. These ‘skirmishes’ included over fifty white youths marching through the market, smashing the windows of Pakistani-owned shops and leaving three Pakistani youths requiring hospital treatment.^62^ Two white youths and two Pakistani youths were arrested, yet only the latter were charged (with carrying an offensive weapon). Despite the concerted efforts of Hunte, Shore, and certain Pakistani political leaders to try to tackle the problem of racist violence through political structures and conventional channels, there was still much resistance to recognising the depth of the problem and its potentially devastating consequences. For example, at the first meeting, it was noted that:

Some people felt that the problem was not one of colour so much as hooliganism by bored and socially deprived youngsters … In general it was felt that too much publicity for skinheads would only encourage them and might lead to similar incidents elsewhere.^63^

It was also argued that it was ‘essential to improve communication between the Pakistanis and the host community’ and to ‘restore Pakistani confidence in the capability of the law enforcing authorities to protect them’. In essence, such discourse diminished the part played by racism in informing such acts of violence and instead directed attention back towards members of the Pakistani community themselves and the need for them to have greater confidence in existing local structures and their capacity to resolve these tensions.

The discussion of the integrationist attempts to address racist violence shows that some degree of consent to the discourse of law and order and the authority of the police was achieved through a coming together of the political establishment, the CCoTH, the police and selected sections of civil society (e.g., local religious leaders, the NFPA and the PWA). However, some Pakistani political leaders were becoming increasingly frustrated with the integrationist response. Laying claim to a formal membership of 30,000 people, the PWA, the NFPA and the Demonstration Committee of Multiracially Committed Pakistani Associations, organised a national rally in London on 24 May 1970 under the banner ‘STOP SKINHEAD HOOLIGANISM, DEMAND EFFECTIVE MEASURES, SUPPORT MULTIRACIAL DEFENCE’.^64^ This rally marked both a departure from what hitherto had been a political repertoire based on meetings, lobbying politicians and letter-writing campaigns, to a growing turn to the ‘politics of the street’. Just a few weeks prior to the march, Lutfur Rahman, Chair of the Stepney PWA, publicly broke with the consensus that it was vital that communication between the local Pakistani population and the so-called ‘host community be improved’:

We want the Home Secretary to issue a statement condemning this sort of thing … The police are just not interested … Repercussions among Pakistanis in Britain would be ‘outside of our control’ if the wave of Paki-bashing was not halted … We are in fear for our children and womenfolk … we love London. We don’t want it turning into a battleground like some American cities.^65^

The organising of the rally and Rahman’s claim that repercussions would be ‘outside of our control’ were most likely a recognition of the challenges posed by the emergence of an autonomous anti-racist approach to the problem of racist violence and harassment.

### The autonomous response to racist violence

As mentioned earlier, 1968 marked in many ways a turning point in race relations. According to Sivanandan:

Blacks by 1968 were beginning to fight as a class and as a people. Whatever the specifics of resistance in the respective communities and however different the strategies and lines of struggle, the experience of a common racism and a common fight against the state united them at the barricades. The mosaic of unities … resolved itself … into a black unity and a black struggle.^66^

Organisations such as the UCPA, the Racial Action Adjustment Society and the Black Panthers became increasingly concerned with challenging fascist violence, police brutality and documenting and fighting the cases of those who had either been framed or beaten up by the police.^67^ At the local level, between 1968 and 1970, a number of black radical, anti-imperialist and socialist groups entered the political field in the East End to support the ‘politics of the street’.^68^ Following Tosir Ali’s murder, newspaper reports noted that the ‘area’s troubles have attracted the militants. Black Panthers, the [UCPA], the Third World Party and the [PWU] … none of which are based in East London.’^69^

Immediately after Tosir Ali’s murder, the PPP and the UCPA distributed leaflets explaining that they had approached Peter Shore MP with sixteen complaints of assaults and robbery on Pakistani people living in the Aldgate East area and that Shore had ‘refused to help us’ as well as noting that the ‘police in the area too have failed[us]’. The leaflet also stated that ‘IF THE LAW CANNOT PROTECT US, THEN WE HAVE THE RIGHT TO DEFEND OURSELVES’.^70^ Another PPP-UCPA leaflet distributed around the same time declared:

Self-defence = No other Choice … We therefore have no choice but to organise ourselves to prevent further racist violence. Our policy will be tit for tat. There will be a tooth for a tooth. Criminal assaults will be met by just retaliation. Racists beware!^71^

The PPP, which claimed to have 275 members, announced that it would also be setting up karate and judo classes in Whitechapel to enable members of the local Pakistani population to defend themselves. Supported by George Joseph of the UCPA, Abdul Hye told the national media that the PPP ‘is a defensive organisation. We have no intention of fighting or killing anyone, but if it comes to us, we will hit back.’^72^ For Hye and his allies, the call for self-defence groups to be formed was a direct response to the failure of the police and the broader political establishment to respond effectively to the entrenched and persistent racist violence in Tower Hamlets:

We have lobbied our MP, Mr. Peter Shore, 15 times about this problem in the East End, culminating in the united demonstration we had to the House of Commons with the UCPA last Monday. We saw Marcus Lipton [Labour MP for Brixton], Peter Shore, and Mr. MacNair Wilson [Conservative MP for New Forest]. The next day [Tosir] Ali was murdered. We have given our documents to Mr. Shore who has promised to take action … We plan to distribute 1,000 leaflets explaining our situation to the British people, and to hold a demonstration. If the authorities still fail to act, we will organise street patrols … We shall have no choice but to organise self-defence.^73^

With little faith in the political establishment, Abdul Issaque of the PWU later argued that it would leave attempts to seek reform to the ‘recognised welfare associations [e.g. the NFPA and the PWA] …They write letters and sit in offices. Our aim is the protection of our people.’^74^ Amidst the calls for self-defence formations, Ekra Huq, president of the Pakistani Student Federation, said that the patrols would be ‘multiracial’.^75^ As the discussion thus far demonstrates, both the integrationist and autonomous anti-racist responses to racist violence lobbied the political establishment, organised public meetings, demonstrations and marches, and collected information that documented the scale of racist violence inflicted upon Pakistani people. However, it was the calls for self-defence that would prove to be the source of tension.

As noted above, PPP and PWU responses to racist violence in Tower Hamlets built upon the multiethnic network of black radicals, anti-racists, anti-imperialists, socialists and some trade unionists forged around the time of Powell’s speech. Immediately after Tosir Ali’s murder, the Chair of the WPPE, Alex Hart wrote to Peter Shore inviting him to a local meeting being organised by the PWU with the support of the BPA and the ‘No.1 Region’ of the Transport and General Workers Union.^76^ On Sunday 19 April, up to 1,000 people attended this meeting where they heard organisers pour scorn on the meeting that Shore, the NFPA and the PWA had held the previous day.^77^ What is more, newspaper reports noted that the NFPA had been branded ‘Uncle Toms’.^78^ The meeting also heard messages of support from Clive Jenkins, the General Secretary of the white-collar union, the Association of Scientific, Technical and Managerial Staff, and Ted Johns, a local Labour councillor in Tower Hamlets. Johns, had in fact, resigned the whip of the local Labour Party claiming that ‘we have got to get on the streets and stop this violence’.^79^ At the end of the meeting, thirty to forty men stepped forward and volunteered to take part in self-defence patrols. Sibghat Kadri, Chairman of the PWU’s legal committee, told the meeting:

Peaceful we may be, but cowards we are not … Pakistanis will not take the law into their own hands, but will adopt self-defence … Even if you kill in self-defence, it will not be murder … Enoch Powell is the godfather of what is happening here.^80^

Throughout April 1970 around 200 people took part in self-defence patrols.^81^

Supported by the Black Panther Movement, the BPA and the INLSF, up to 2,000 people marched from Speakers Corner in Hyde Park to Downing Street on Sunday 3 May 1970.^82^ During the march, Edward Davoren of the INLSF told reporters that ‘the Pakistani cause was the same as the Irish cause’ and that he hoped ‘to form Irish-Pakistani defence groups in East London’.^83^ Before the rally set off, speeches connected the local struggle against racist violence and harassment to broader international struggles, claiming that: ‘Racialism was a function of capitalism and demanding that Pakistanis see their fight as an aspect of the world struggle of oppressed classes.’^84^

Once marchers were on their way to Downing Street, the police re-routed the demonstration so as to avoid clashes with the Monday Club – a far-right anti-immigration organisation affiliated to the Conservative Party but which also drew support from members of the NF. Despite the re-routing, Monday Club supporters attacked the march shouting ‘go home’ and ‘Down with the Reds’.^85^ As the march passed the Irish Embassy, demonstrators chanted ‘British Imperialism – Out, People’s War – Yes’, ‘Black Power, People’s Power’, ‘All Unite – Black and White’ and ‘Disembowel Powell’.^86^ Once the demonstration reached Downing Street, Abdul Issaque of the PWU submitted a memorandum outlining that ‘every peaceful method to relieve their suffering’ had been pursued and that they had ‘reached the point where they realise that the only answer lies in self-defence for their safety and welfare’.^87^ Issaque, a ‘self-confessed Marxist’, also claimed that:

I do not believe it is only skinheads who are causing this violence. There are fascists behind it all, and they are organised … These are Powellites, National Front supporters and National Socialists. These are different groups but their aims are the same – to keep Britain white … we have no faith in the Metropolitan Police. Many of them are racialist and they are allowing atrocities to go on. Callaghan refuses to meet us face-to-face and discuss this.^88^

Though there were, as shown above, areas in which both the integrationist and autonomous approaches used the same tactics, they both followed long-established patterns of seeking recourse through official channels, petitions and letter writing to local and national state officials. They organised public meetings, demonstrations and marches, and collected information on racist attacks (because of the failure of the police to act) in order to lobby the Home Secretary and local MPs. But they held very different views on the police and the state. By the spring of 1970, the PWU and the PPP had shifted away from trying to engage the state, coming to see its institutional apparatuses, such as the CCoTH and the police, as ineffectual in the battle against racist violence and harassment. Alongside their allies, they openly countered the integrationist approach, contending that the racist state could not be reformed.

Whereas the integrationists sought to defend varying aspects of the state, the alliance of Black Power and anti-imperialist anti-racists sought to tackle both the dominant values and apparatuses of law and order and challenge the established forms of Pakistani leadership in local civil society. In contrast to the integrationist approach, the PPP and the PWU articulated a more sophisticated understanding of the nature and scale of wider societal racism. In so doing, they demonstrated how ‘Powellism’, the 1968 Commonwealth Immigrants Act, fascist political agitation and everyday racist violence and harassment were interconnected by a shared desire to keep Britain white. However, the call to form self-defence squads was seen by both liberal and conservatives as inflammatory and was widely condemned by those who had coalesced around the so-called ‘moderate’, ‘peaceful’ integrationist approach.

For Peter Shore, the PPP and the PWU were simply ‘making the situation worse’, while the NFPA claimed that Abdul Hye of the PWU was ‘irresponsible and dangerous’.^89^ Joe Hunte, Chair of the CCoTH, argued that ‘We don’t need vigilantes … we don’t need stirrers’,^90^ and, like many others, he also sought to position those arguing for self-defence tactics as ‘outsiders’:

Pakistani tough-liners are coming in from outside Tower Hamlets … They come from North London … We don’t want trouble … I can’t find a single vigilante-type in my whole area … It’ll be over my dead body. Self-defence is one thing. But these people talk about more than that … If you get a group of Pakistanis who want to oppose a so-called group of attackers, you’re going to get gang warfare.^91^

As well as stating his opposition to ‘vigilante groups patrolling the streets’ on religious grounds, Mukhta Ahmad Mir, former president of the PWA, opposed the socialist politics of the PPP and the PWU:

there are dangers when those with political ambitions start organising people … I am speaking on behalf of Moslem Pakistanis, and our religion is a powerful weapon against communism.^92^

Similarly Raja Mahmudabad, President of the League of Overseas Pakistani Organisations called for a ‘multiracial approach’, warning that:

In such a situation where racial prejudices are allowed to pervert the vision and fanaticism and false militancy [are] allowed to run riot … such a course [i.e. self-defence patrols] is also alien to the spirit and practice of Islam … [The Pakistani community] should never invite or provoke attacks by an ostentatious display of force.^93^

In addition to facilitating the debate between the integrationist and autonomous approaches, sections of the national print media were also critical of those mobilising around a black political identity. For example, an editorial in the *Daily Mail* posited that ‘Black Power will only breed white resentment … The role for all of us, black and white, is to work together.’^94^

In summary, the rebuttal of self-defence tactics was a feature of how law and order came to be understood and experienced through race. For some integrationists, both self-defence measures and the philosophy of socialism that underpinned it were deemed incompatible with a Pakistani identification. At the same time, they, along with parts of the right-wing press, denied the multiethnic basis of the autonomous approach while making their own pleas for multiracial unity. By framing Black Power, the PPP and the PWU as extremist outsiders, these attempts to condemn self-defence tactics also sought to reassert the authority of the NFPA, the PWA, the local MP, the police and the CCoTH. Moreover, the view that self-defence tactics would only risk further violent disorder shifted the focus away from police ambivalence and the failure of the state to halt rising levels of racist violence and harassment. To some extent, the integrationists considered ‘vigilantism’ to be the more immediate and pressing social problem as they attempted to delegitmise self-defence as a political act by arguing that it breached the dominant values of law and order and the authority of the police. In this sense, the term ‘vigilantism’ was used to translate a political issue into a criminal one, thereby making it easier to uphold the liberal order while at the same time imposing legal sanctions (e.g., arresting the victims of racist violence and harassment when acting in self-defence). In short, self-defence was delegitimised as mindless violence.

## Conclusion

1968 was not only a time when Powell’s speech provided a lightning rod that sparked an escalation in racist violence, it also marked the moment when Black and Asian politics were profoundly transformed by anti-colonial struggles and the Black Power movement in the United States. Together, these developments transformed how some racialised minorities combated racist violence in Britain.

In a place like Tower Hamlets, typified by heightened violence, the integrationist approach brought together the local CRC (CCoTH), the local Labour MP and civil society organisations such as the NFPA and the PWA. Using conventional political campaigning methods, they sought to make the political establishment, the local state and parts of civil society more aware of just how perilous everyday life had become for the local Pakistani population. Yet at the same time, the integrationist approach obfuscated the racism of the political class, bolstering official and public interpretations of racist violence as being the pastime for an immoral, ‘irresponsible’ and lunatic fringe.

Those taking the ‘autonomous’ approach fused Black Power, anti-imperialist and socialist ideas, to create a multiethnic alliance of African, Asian, Caribbean and Irish people. Such an approach offered a more sophisticated understanding of the nature and scale of racist violence and harassment. Attentive to the impact of Enoch Powell’s ‘Rivers of Blood’ speech, fascist political agitation and racist immigration controls, the PPP, PWU and their allies looked to overcome the failure of the police and the political establishment to take appropriate action against racist violence by advocating for the politics of self-defence. In response, the integrationists tried to shore up consent for the police and the dominant values of law and order by condemning the PPP, PWU and their allies as ‘extremist outsiders’ and by recasting, if not criminalising, the politics of self-defence as mindless violence. Recovering this ‘hidden history’ of Pakistani responses to racist violence between 1968 and 1970 is significant because this period saw Black Power organisations standing alongside other anti-colonialists and socialists to forge autonomous and vibrant ‘communities of resistance’. In doing so, they bequeathed a legacy of militant collective anti-racism.
